# Contributions to the systematics of New World macro-moths VII

**DOI:** 10.3897/zookeys.788.30148

**Published:** 2018-10-08

**Authors:** B. Christian Schmidt, J. Donald Lafontaine

**Affiliations:** 1 Canadian National Collection of Insects, Arachnids, and Nematodes, Ottawa Research and Development Centre, Agriculture and Agri-Food Canada, KW Neatby Bldg., 960 Carling Ave., Ottawa, Ontario, Canada K1A 0C6 Canadian National Collection of Insects, Arachnids, and Nematodes Ottawa Canada

This special issue of ZooKeys is the seventh volume in the “*Contributions*” series, dedicated to disseminating systematics research on the Noctuoidea, Geometroidea, and other macro-moth groups. Previous volumes were published in May 2009 (volume I, ZooKeys # 9), March 2010 (volume II, ZooKeys # 39), November 2011 (volume III, ZooKeys # 149), February 2013 (volume IV, ZooKeys # 264), June 2014 (volume V, ZooKeys # 421) and October 2015 (volume VI, ZooKeys # 527; see Schmidt and Lafontaine 2009, 2010, 2011, 2013, 2014, 2015). Authors interested in contributing to future “*Contributions*” volumes are encouraged to contact us.

In the current volume, eighteen authors contributed eleven manuscripts on New World taxa in the Erebidae, Noctuidae and Apatelodidae. In addition to numerous taxonomic and nomenclatural changes, 16 new taxa are described from the Nearctic, including 1 new genus, 14 new species and one subspecies: *Hypoprepialampyroides* Palting & Ferguson, **sp. n.**, *Clemensiaochracea* Schmidt & Sullivan, **sp. n.**, *Euchaetesnancyae* Nagle & Schmidt, **sp. n.**, *Dolocuculliapoolei* Crabo & Hammond, **sp. n.**, *Sympistiseleaner* Adams, **sp. n.**, *Sympistisferrirena* Crabo, **sp. n.**, *Aseptisharpi* Crabo & Mustelin, **sp. n.**, *Admetovisicarus* Crabo & Schmidt, **sp. n.**, *Rhabdorthodes* Crabo, **gen. n.**, *Rhabdorthodespattersoni* Crabo, **sp. n.**, *R.petersoni* Crabo, **sp. n.**, *Hypotrixlactomellis* Wikle & Crabo, **sp. n.**, *Plagiomimicusyakama* Crabo & Wikle, **sp. n.**, *P.yakamamojave* Wikle & Crabo, **ssp. n.** and *Plagiomimicusincomitatus* Mustelin, **sp. n.**

An additional eight species are described from the Neotropics: *Apatelodesnavarroi* Herbin & Beccacece, **sp. n.**, *Apatelodeschalupae* Herbin & Beccacece, **sp. n.**, *Apatelodesulfi* Herbin & Beccacece, **sp. n.**, *Lophocampaazuayensis* Vincent, **sp. n.**, *Lophocampacarpishensis* Vincent, **sp. n.**, *Leucosigmasolisae* Goldstein, **sp. n.**, *Leucosigmapoolei* Goldstein, **sp. n.** and *Leucosigmaschausi* Goldstein, **sp. n.**

All updates, additions and corrections to the Check List of North American Noctuoidea (Lafontaine and Schmidt 2010) since the last update in 2015 are summarized.
